# Immunofluorescence Tomography: High-resolution 3-D reconstruction by serial-sectioning of methacrylate embedded tissues and alignment of 2-D immunofluorescence images

**DOI:** 10.1038/s41598-018-38232-9

**Published:** 2019-02-13

**Authors:** Geraint J. Parfitt

**Affiliations:** 1 European Cancer Stem Cell Research Institute, Cardiff University, Maindy Rd, Cardiff, Wales United Kingdom; 2 School of Optometry & Vision Sciences, Cardiff University, Maindy Rd, Cardiff, Wales United Kingdom

## Abstract

Immunofluorescence tomography is a high-resolution 3-D reconstruction method based on methacrylate embedding and serial-sectioning, where 2-D images of immuno-stained serial-sections are computationally aligned into image stacks, and the 3-D volume rendered. Butyl-Methyl Methacrylate (BMMA) plastic was adopted as it preserves excellent tissue morphology and can be de-plasticized easily using an organic solvent, which enables immuno-staining of serial-sections without antibody penetration issues over millimeters of 3-D reconstructed tissue (Z-depth). High axial Z-resolution over a large volume was achieved by cutting serial-sections at 2 µm thickness. Stained sections were imaged by multiple modalities, including immunofluorescence, electron microscopy and second harmonic generation (SHG), and there are advantages over confocal microscopy as the tissue does not need to be cleared, while antibody penetration or light scattering issues are minimized. The plastic serial-sections can be re-probed, without a loss in tissue structure, using low pH glycine hydrochloride antibody elution. It is a cost-effective approach as the microscopes needed are significantly cheaper than confocal microscopes and sections can be kept indefinitely. Therefore, immunofluorescence tomography is a powerful new tool to quantify sub-populations of cells in high-resolution 3-D using antibody fluorescence. This article describes the immunofluorescence tomography method for 3-D reconstruction of epithelial tissues such as mammary gland, cornea and the hair follicle.

## Introduction

Fluorescent antibody labelling of tissues is a fundamental tool used in the laboratory to visualize the three-dimensional distribution of multiple proteins within a biological sample.

Immunofluorescence microscopy based 3-D reconstruction is an area of intensive research and recent methods have significantly improved resolution by modifying tissue preparation, including expansion microscopy^1^, CLARITY^2^, iDISCO^3^ and array tomography^4^. However, each approach has their own advantages and limitations when generating high-resolution 3-D reconstructions of fluorescent signals. For example, confocal microscopy based 3-D reconstruction methods, which rely on a pinhole to eliminate out-of-focus light, are limited by the working distance of the objective lens; require chemical clearing of the tissue for optical transparency; and antibody penetration and background fluorescence are problematic in larger specimens. Immunofluorescence tomography generates 3-D reconstructions of fixed tissues at high-resolution by serial-sectioning of butyl-methyl methacrylate (BMMA) plastic embedded specimens. Here, the principles and applications of immunofluorescence tomography are outlined for multiple tissues to demonstrate how the limitations of the current 3-D reconstruction approaches can be overcome with this new method.

The excitation wavelength of light and the numerical aperture of the objective determine lateral (X-Y) resolution. Light scattering that compromises resolution in a large sample can be removed by optically clearing tissues and reducing refractive index differences, which has enabled visualization of deep tissue structures at high-resolution. Whole-mount immuno-labelling approaches that use tissue clearing for high-resolution 3-D reconstruction include ClearT2^5^, 3DISCO^6,7^, SeeDB^8,9^, CLARITY^2^ and CUBIC^10^. However, tissue clearing can be time consuming for larger samples and antibody staining limited, particularly when denaturing agents such as urea or SDS are used. Alternatively, light scattering can be completely removed to improve resolution through physical sectioning of BMMA plastic-embedded tissues at 2 µm or less. Serial-sections cut at 2 µm thickness enable 3-D reconstructions of larger tissue volumes to be generated as less sections need to be cut and, importantly, each cell nuclei falls within the focal plane using a 20X/0.75 objective lens. As the axial resolution of 3-D reconstructions is determined by the physical thickness of the serial-sections, high-resolution reconstructions (0.1 µm–2 µm Z-resolution) can be generated by cutting less thick serial-sections and acquiring images by multiple modalities, such as immunofluorescence, electron microscopy, second harmonic generation (SHG) and two-photon excitation fluorescence (TPEF). Methacrylates were originally designed for embedding tissues for electron microscopy, so this technique enables correlative microscopy imaging with the different wavelengths used in fluorescence and electron microscopy.

BMMA plastic embedding and mosaic imaging of immuno-stained serial-sections is achievable with a standard fluorescence microscope, which means that immunofluorescence tomography is a cost-effective approach to high-resolution 3-D reconstruction compared to confocal microscopy approaches. BMMA plastic-embedded tissues also retain excellent morphological preservation and the plastic can be removed from the tissue after sectioning using an organic solvent (i.e., acetone). This enables immuno-staining of semi-thick sections (<2 µm) for high-axial resolution images without any antibody penetration issues. Other embedding plastics, such as LR white, are typically resistant to de-plasticization by an organic solvent so antibody staining is limited to serial-sections under 200 nm thick^4^, which means that 3-D reconstruction of tissues in the millimeter range is not realistic. Importantly, ultra-thin plastic sections can be immuno-stained and eluted of their antibodies using low pH glycine hydrochloride or SDS, for sequential immuno-labelling steps that is not possible with tissue clearing and confocal 3-D reconstruction methods^4^. Background fluorescence from antibodies and auto-fluorescent signals is reduced in ultra-thin sections compared to thick tissue sections as unbound antibodies are washed away easier and fluorescent signals are confined to the focal plane of each serial-section. Paraffin and cryo-embedding methods are routinely used for high-throughput sectioning in clinical histology, but these soft embedding mediums cannot be cut under 5 µm; sections cut thicker than 2 µm exhibit nuclei within different focal planes, which significantly affects the resolution of 3-D reconstructions. This results in compression artefact when using soft embedding mediums, so they are not viable options for 3-D reconstruction of tissue and are more advantageous to high-throughput 2-D studies, such as diagnostic imaging.

This article advances on the first studies that used BMMA plastic serial-sections for 3-D reconstruction of antibody fluorescence^11^, to establish the immunofluorescence tomography method for any fixed tissue, including the adult mouse mammary gland; colon; cornea; developing forelimb at embryonic day 15.5; and calcified mouse bone samples. Furthermore, it details a more cost-effective protocol for antigen retrieval and immuno-staining when generating high-resolution 3-D reconstructions. Finally, it is detailed how to perform image acquisition by multiple imaging modalities for 3-D reconstruction, and how it is possible to quantify sub-populations of cells based on fluorescence intensity of endogenously expressed fluorescent proteins or immuno-labels.

## Results

**Figure 1:**
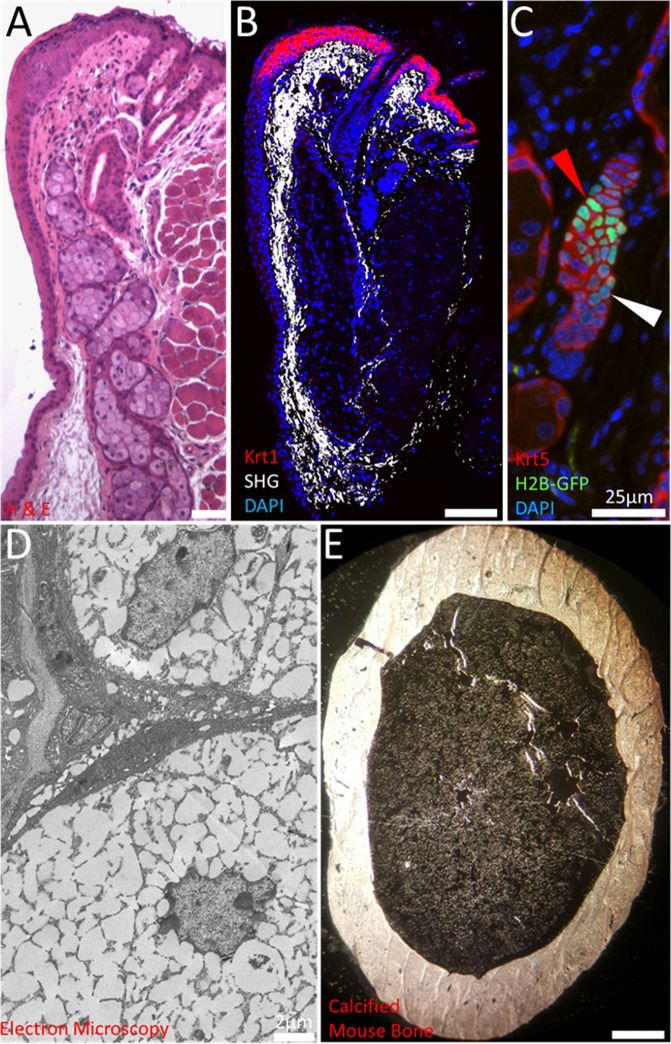
BMMA plastic sections can be immuno-labelled and imaged using multiple imaging modalities, such as non-linear optics. (**A**) Histological stains hematoxylin and eosin can be used to outline basic cell and tissue structures, such as muscle and hair follicles of the eyelid. (**B**) Immunofluorescence staining of proteins (keratin 1 – red) and second-harmonic imaging of collagen fibers (white) enables probing of protein expression and extra-cellular matrix structure, with cell nuclei distribution identified by DAPI staining (blue). (**C**) Endogenously expressed fluorescent proteins, such as the histone H2B-GFP fusion protein in the H2B-GFP/K5tTA mouse, were imaged for fluorescence intensity analysis of GFP and co-localization with antibody stained proteins, such as keratin 5 (red). (**D**) BMMA plastic sections cut at 100 nm were contrasted with heavy metal staining (uranyl acetate and phospho-tungstic acid) and imaged using a JOEL 1010 transmission electron microscope. Differentiated Meibomian gland cells undergoing lipogenesis and holocrine secretion are readily visible. (**E**) Darkfield micrograph of a BMMA section of calcified mouse femur. (Scale bars: A–C 100 µm; D 2 µm; E 100 µm).

Eyelids from wild-type and H2B-GFP/K5tTA bi-genic mice were fixed in 2% PFA and embedded in BMMA plastic for serially-sectioning at 2 µm and imaging by multiple imaging modalities. Mouse eyelid sections were stained for hematoxylin and eosin (H&E) first, to verify the compatibility of BMMA embedding with basic histological stains (Fig. 1A). Next, BMMA sections were imaged by second harmonic generation (SHG) to visualize fibrillar collagen in the eyelid. The SHG signal from collagen was overlaid with anti-keratin 1 immuno-labelling to mark epidermis (Fig. 1B).

To confirm the BMMA plastic embedding process retains endogenous fluorescence proteins such as GFP, we imaged keratin 5^+^ label-retaining cells (LRCs) in the H2B-GFP/K5tTA mouse hair follicle after pulse-chase (Fig. 1C). The improved axial resolution afforded by sectioning tissues at 2 µm meant that fluorescence intensity differences were observed according to high (red arrow) or low (white arrow) GFP expression, which indicated a range of label-retention and quiescence. BMMA serial-sections cut at 100 nm were mounted onto grids and stained with heavy metals (uranyl acetate and phospho-tungstic acid) for contrast when using the transmission electron microscope, where cells undergoing lipogenesis and holocrine secretion were visible (Fig. 1D). It is also possible to embed mouse calcified bones in BMMA for serial-sectioning (Fig. 1E).

**Figure 2:**
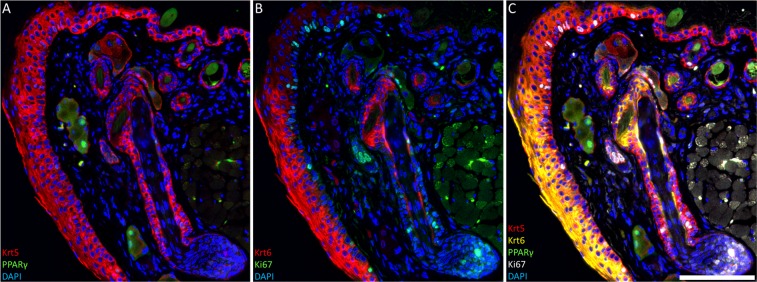
Sequential immuno-staining of 2 µm BMMA sections of mouse eyelid. (**A**) Keratin 5 (red) is expressed in the basal epithelia of the skin and conjunctiva, while Pparγ (green) is localized to differentiated cells undergoing lipogenesis and holocrine secretion. (**B**) After antibody elution using glycine hydrochloride (pH2.5) at 55 °C, the same section was stained for keratin 6 (red) and Ki67 (green), to label conjunctiva and actively dividing cells, respectively. (Scale bars = 100 µm).

To evaluate elution and re-probing of antibodies for sequential immuno-staining, glycine hydrochloride (pH 2.5) was used to elute keratin 5 and peroxisome proliferating activating receptor-gamma (PPARγ) primary antibodies from eyelid BMMA sections for further re-probing (Fig. 2A). PPARγ marks cells undergoing lipogenesis in the sebaceous glands of the eyelid and hair follicle. By re-probing the sections with anti-keratin 6 and -Ki67 immuno-staining (Fig. 2B) and aligning them using DAPI, it was possible to image more than 4 fluorescent channels, which is the conventional limit. Keratin 6 was localized to the eyelid conjunctiva and hair follicles, while Ki67 labelled actively dividing cells. Though this approach, DAPI staining and 4 primary antibody probes were visualized on the same eyelid section (Fig. 2C).

**Figure 3:**
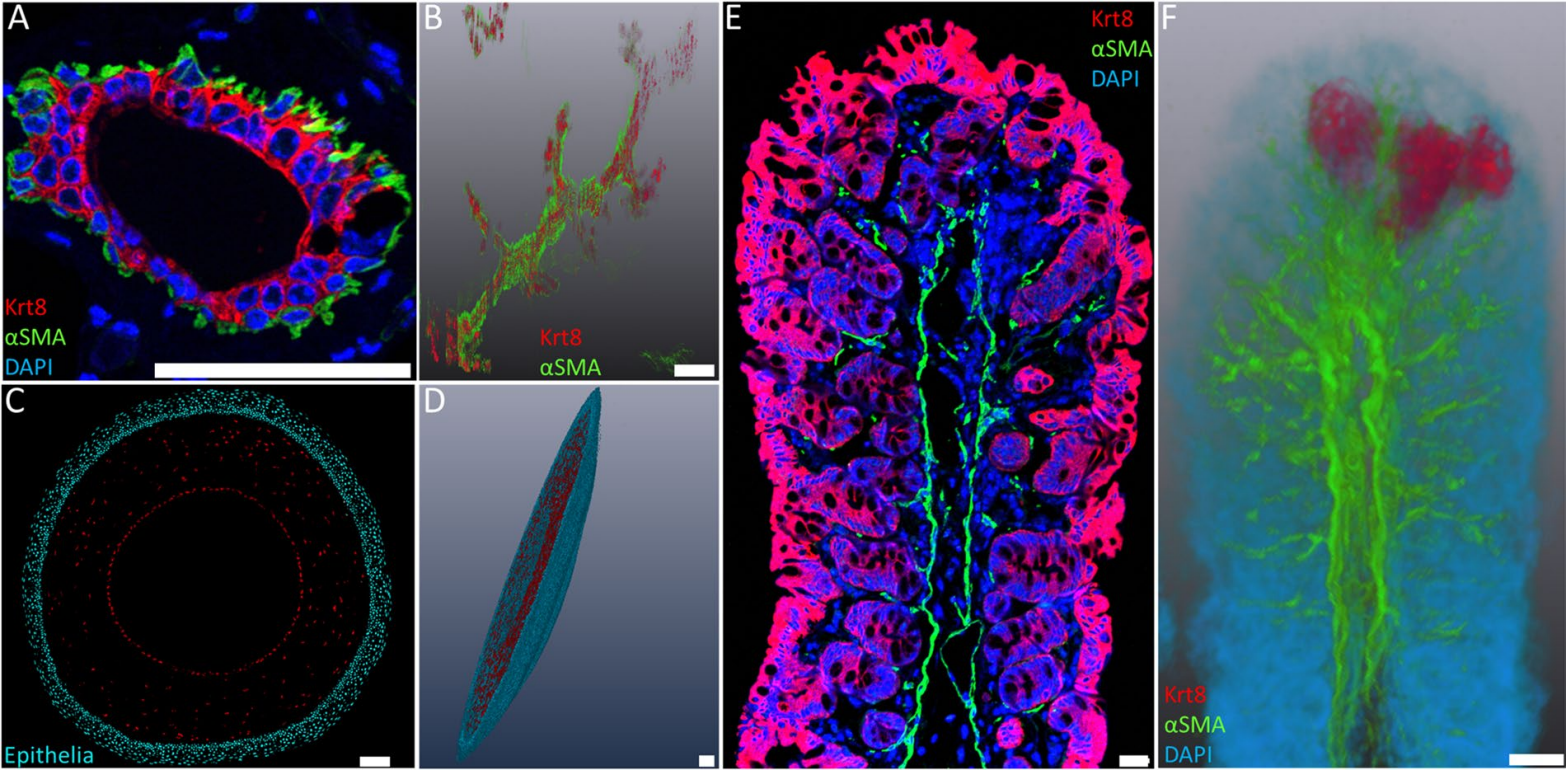
High-resolution 3-D reconstructions of epithelial tissues generated by immunofluorescence tomography. (**A**) Mouse mammary gland luminal layer stained with keratin 8 (red) and basal layer stained with α-SMA (green). (**B**) 3-D reconstruction of keratin 8 and α-SMA immuno-staining of the mouse mammary gland. (**C**) Mouse corneal epithelium, underlying stroma and endothelium. (**D**) Cornea 3-D reconstruction using DAPI staining of epithelial cells (cyan) and keratocytes/endothelium (red). (**E**) Mouse colon stained for keratin 8 (red) and α-SMA (green). (**F**) Immunofluorescence tomography 3-D reconstruction of the mouse colon, with individual villi segmented. (Scale bars = 100 µm).

**Figure 4:**
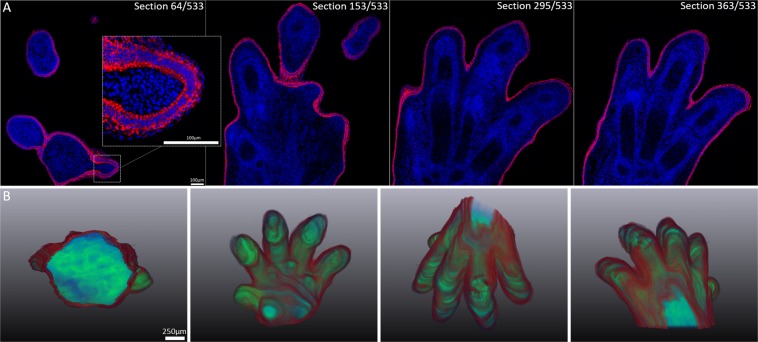
3-D reconstruction of the developing mouse forelimb at embryonic day 15.5. (**A**) Keratin 5 immuno-staining was localised to the superficial epithelium. (**B**) The 3-D reconstruction of the whole mouse forelimb was generated from 533 serial-sections (1066 µm z-depth) cut at 2 µm and imaged using a 20X/0.75NA objective.

After immuno-staining of 2 µm BMMA serial-sections, generation of high-resolution 3-D reconstructions of tissues, such as mammary gland, colon, cornea and the developing forelimb was possible by alignment of serial-sections using Amira software (Fig. 3). In the mammary gland, keratin 8 (red) labels the inner luminal cells, whereas contractile myoepithelial cells in the basal layer are positive for α-smooth muscle actin ((α-SMA) - green) (Fig. 3A). The mammary gland 3-D reconstruction of keratin 8 and α-SMA in Fig. 3B is comprised of 379 aligned serial sections (758 µm Z-depth). To 3-D reconstruct the entire mouse cornea with the epithelial and keratocyte population segmented separately, 97 serial-sections (194 µm Z) were imaged using DAPI staining (Fig. 3C,D). In the mouse large intestine, keratin 8 (red) labels the lining of the gut epithelium and the vasculature is marked by α-SMA (green) immuno-staining of blood vessels (Fig. 3E). The colon 3-D reconstruction, with segmented villi (red) was generated from 170 sections or 340 µm in Z-depth (Fig. 3F). The developing forelimb was 3-D reconstructed at embryonic day 15 from 533 serial-sections (1066 µm Z-depth). High-resolution 2-D images from the sequence of serial-sections were acquired as an 8 × 8 mosaic using tile scanning with the Leica DMI6000B (Fig. 4A). Keratin 5 (red) marked the entire basal epithelium of the epidermal layer that forms the surface of the developing mouse forelimb, as seen in 3-D reconstruction of the entire forelimb (Fig. 4B).

**Figure 5:**
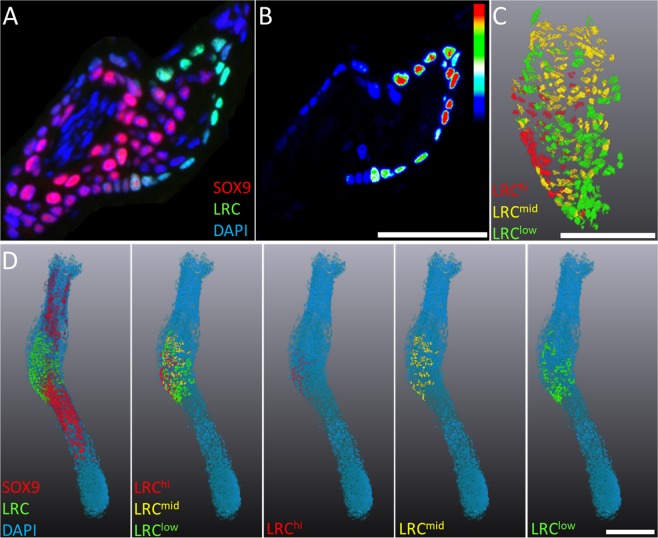
Fluorescence intensity gradients in 3-D. (**A**) Because of the improved axial resolution afforded with immunofluorescence tomography, fluorescence intensity gradients of immuno-stained proteins and endogenously expressed fluorescent proteins can be visualized. (**B**) Label-retaining cells in the hair follicle of the H2B-GFP/K5tTA mouse retain GFP label according to their cell cycle rate - the most quiescent cells are the strongest label-retaining cells. (**C**) 3-D reconstruction of label-retaining cells in the mouse hair follicle bulge compartment, colored by increasing fluorescence intensity and label-retention (Green > Yellow > Red). (**D**) Immunofluorescence tomography 3-D reconstruction of the mouse hair follicle, with SOX9 expression and label-retaining cells shown according to fluorescence intensity. (Scale bars = 100 µm).

Fluorescence intensity gradients of endogenously expressed fluorescent proteins and immuno-labels were seen as a result of the improved axial resolution afforded by immunofluorescence tomography (Fig. 5). Here, variable expression of SOX9 and GFP was seen across cells of the mouse vibrissae hair follicle (Fig. 5A). GFP retention in label-retaining cells of the H2B-GFP/K5tTA mouse was qualitatively assessed through thresholding of the LRC fluorescence between 0 to 255 from an 8-bit image (Fig. 5B). The slowest cycling cells which retained GFP label the strongest were pseudo-coloured red (LRC^hi^ = 200+) and represented 22% of the total LRC population; the next population of weaker LRCs constituted 34% of LRCs and were graded from 100–200 (LRC^mid^ yellow). The weakest LRCs were pseudo-coloured green (LRC^low^ = 0–100) and were quantified as 44% of the total hair follicle LRC population (Fig. 5C). From the 3-D reconstruction of the hair follicle in Fig. 5D, we quantified a total of 654 total DAPI cell nuclei, of which, 91 were LRCs (20 LRC^hi^; 31 LRC^mid^; and 40 LRC^low^). This suggests that label-retaining cells vary in their number of cell divisions and illustrates how the expression of immunofluorescent markers could be assessed in cells that range in quiescence. Finally, the fluorescence intensity index was visualised three-dimensionally and co-localized with SOX9 expression to show how slow-cycling stem cells are confined to the hair follicle bulge, paticularly the most quiescent cells in red, which are candidates for the true stem cell population (Fig. 5D).

## Discussion

Immunofluorescence tomography is a powerful tool for volumetric analysis of tissues using multiple imaging modalities (e.g., immunofluorescence, electron microscopy, SHG and TPEF), which retains excellent morphological preservation. We have shown here that immunofluorescence tomography is a powerful approach to grade fluorescence intensity in 3-D and reliable volumetric analysis is possible as morphology is well preserved. Using the H2B-GFP/K5tTA mouse originally developed to image epidermal stem cells^12^, we were previously able to identify LRCs in the meibomian gland and determine the range of intensity differences between LRCs in the hair follicle^13,14^. Multiple cell sub-populations can be quantified in 3-D according to the expression of immuno-stained proteins markers as well as endogenously expressed proteins.

This paper outlines the immunofluorescence tomography technique and how it can be applied to 3-D reconstruct a wide-range of fixed tissues, including mouse mammary gland, colon, hair follicle and limbs in development. 3-D analysis of lineage tracing in mouse models is useful to determine the cell of origin of tissues and has already been used with immunofluorescence tomography to elucidate the turnover and existence of unipotent progenitors in the eyelid meibomian gland^14^. Endogenous fluorescent proteins such as GFP are preserved during BMMA embedding, however if they are quenched, as is the case with RFP, they can be recovered with an appropriate antibody. In the future, it is anticipated that immunofluorescence tomography will be used to investigate label-retention and lineage tracing mouse models to accurately determine the cell cycle dynamics and disease states in an array of epithelial tissues and tumors.

Axial resolution is improved on confocal based 3-D reconstructions techniques and a larger 3-D reconstruction volume can be generated with multiple probing of *in situ* antigens in a sequential manner. The 3-D reconstruction volume is determined by the number of sections cut, so it is possible to generate reconstructions in the millimeter range with sub-micron resolution. While single-plane illumination microscopy (SPIM) can generate larger 3-D reconstructions than confocal microscopy by using a sheet of light to overcome field of view and working distance limitations, it also has the benefit of being perfectly aligned through optical sectioning^15^. However, there remains the possibility of stripe artifacts caused by obstacles to the light-sheet, as sample illumination is typically from one side. Therefore, physical sectioning of tissues can be more beneficial for 3-D reconstruction, particularly because repetitive antibody staining is less challenging, higher resolutions can be achieved as there is no light scattering, or clearing of tissue needed, and optical aberrations are minimized. However, the greater the volume of tissue cut, the more labor-intensive sectioning, imaging and 3-D reconstruction will become.

BMMA is a versatile polymer that can produce a very hard block, methyl methacrylates have previously been used for embedding tissues as hard as bone and imaging LacZ expression^16,17^. Conveniently, it is possible to elute primary and secondary antibodies from BMMA sections by washing sections in glycine hydrochloride pH 2.5 for 1 hr at 55 °C. This is enough to remove antibody staining such as Ki67, which is confined to cell nuclei, however, cytokeratins are abundantly expressed across epithelial tissues and may require further washing. Increasing the temperature of glycine hydrochloride can increase the stripping and will permanently remove DAPI staining from BMMA tissue sections.

3-D reconstruction volumes generated by immunofluorescence tomography are limited by the dimensions of the diamond knife cutting serial-sections. The current maximum is 8 mm in diameter, by using the HistoJUMBO knife by DiAtome. Furthermore, the maximum reconstruction volume is limited by computational limits. For example, a 3-D reconstruction of the entire mouse eye was generated from 1,280 images (4 × 3 mosaic), which is over 2,560 µm and required a minimum of 74GB of RAM to 3-D render the eye volume, so it is unrealistic for most high-end computers currently available. Improving the image acquisition speeds and data management for 3-D reconstruction of large tissue volumes is necessary for immunofluorescence tomography to advance as a technique. For instance, 3-D reconstruction of most human tissues would require a larger diamond-tipped blade for bigger sections and a larger random-access memory capacity to render the 3-D reconstruction.

In conclusion, immunofluorescence tomography is an advanced approach to 3-D reconstruct fixed tissues with unprecedented detail. It has significant benefits in comparison to other 3-D reconstruction approaches, through physical sectioning and sequential immuno-staining with glycine hydrochloride antibody elution. Plastic serial-sections have high axial resolution, which is determined by section thickness (<2 µm), excellent morphological preservation and few imaging artefacts, such as those generated from light scattering and background fluorescence within a whole-mount tissue volume. These advantages provide large volume 3-D reconstructions with high-resolution, where cell sub-populations can be quantified based on their expression of endogenous or fluorescently-labelled proteins, and their intensity can be determined to provide an index of relative expression. This has already been achieved to determine cell cycle rates in the stem cell niches of the meibomian gland, hair follicle and cornea. Immunofluorescence tomography is an extremely cost-effective option for 3-D microscopy as only a standard fluorescence microscope is required, however, an optimized system for multi-dimensional image acquisition with high speed and resolution is desired to generate meaningful 3-D reconstructions in a timely manner. Therefore, immunofluorescence tomography is an extremely valuable new tool to generate *in situ* 3-D structural information with visualization of protein distribution and quantification of cell sub-populations.

## Materials and Methods

### Butyl-Methyl Methacrylate (BMMA) formulation and embedding

H2B-GFP/K5tTA and wild-type (FVB/NJ) mice were bred and maintained at the Heath Park facility at Cardiff University and experimental protocols carried out were approved by the Cardiff University Animal Welfare and Ethics Review Board (AWERB) and the United Kingdom Home Office under a project license (30/3002) to Professor Matt Smalley. All animals were maintained according to the Animals (Scientific Procedures) Act 1986 and they were euthanized by two schedule one methods (i.e., CO_2_ asphyxiation and cervical dislocation). After 28 days pulse of H2B-GFP/K5tTA mice, keratin 5 epithelial cells express the histone H2B-GFP protein so that the nucleus fluoresces green. When doxycycline is included in the mouse diet (2 g/kg) over 28 days, these cells lose 50% GFP fluorescence intensity with every division. This means that slow-cycling stem cells retain GFP label after long-term 28 days chase (LRCs – Label-retaining cells), whereas actively cycling cells lose fluorescence. Therefore, the mice used here were euthanized at P56 (28 days chase).

**Figure 6:**
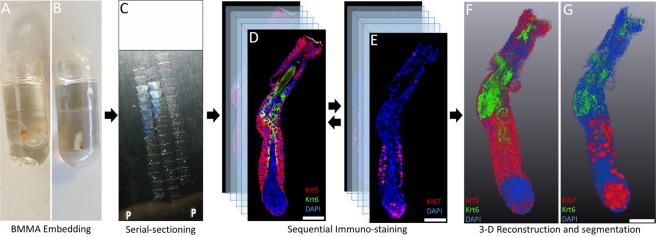
Immunofluorescence tomography method. (**A** and **B**) Tissues were fixed in 2% paraformaldehyde and embedded in butyl-methyl methacrylate plastic (BMMA) under UV light after dehydration and resin infiltration. (**C**) BMMA embedded tissue was serially-sectioned at 2 µm using a diamond knife with an ultra-microtome. (**D**) Serial-sections were immuno-stained, and images acquired using a Leica DMI6000B fluorescence microscope. (**E**) Antibodies were eluted using low pH glycine hydrochloride before re-probing with new antibodies. (**F** and **G**) Serial-section images were aligned using Amira software for 3-D reconstruction, quantification of sub-populations of cells and segmentation of regions of interest.

The general protocol for immunofluorescence tomography, from embedding through to serial-sectioning, immuno-labelling, image acquisition and 3-D reconstruction, is outlined in Fig. 6. Immuno-labeling of butyl-methyl methacrylate (BMMA) sections was first outlined for plant microtubules, in this paper, we describe how it can be used for serial-section and 3-D reconstruction of fluorescent signals at high-resolution. BMMA plastic can be used to embed tissue for thin or thick sectioning and block hardness can be changed according to the ratio of butyl:methyl methacrylate (Sigma Aldrich, St. Louis, MO) and a ratio of 1:4 is suitable for soft tissues and bone samples. While making BMMA, 0.3% Benzoin ethyl ether (BEE) is added to act as a UV polymerization catalyst, while 10 mM dithiothreitol (DTT) is used to limit the effects of free radicals generated during embedding. The final BMMA solution containing BEE and DTT is degassed using nitrogen gas (N_2_) for 30 minutes to remove any remaining oxygen that inhibits polymerization of BMMA. BMMA solution is then stored at −20 °C away from light.

Prior to BMMA plastic embedding, tissues fixed in 2% paraformaldehyde for at least 24hrs were embedded in 3% low melting point agarose to correctly orientate samples. Agarose-embedded tissues were then dehydrated in 50%, 75% and 95% ethanol for 30 minutes each, before a final wash in 100% ethanol for 30 minutes (X3). After dehydration of samples, resin infiltration with BMMA was carried out at a 1:2 (BMMA:ethanol) dilution for 12 hours, then 1:1 and 2:1 BMMA:ethanol dilutions for >12 hours each. Finally, samples were immersed in 100% BMMA overnight before polymerization in size 00 gelatin capsules under UV light at 4 °C. Polymerized BMMA blocks were then ready to be sectioned and immuno-labeled.

### Serial-sectioning of BMMA embedded tissues

BMMA blocks were trimmed with a razor blade into a trapezium-shaped block face around the embedded tissue, with parallel top and bottom edges to help form straight ribbons. Craft glue (Pattex – Henkel, Germany) was applied to the top and bottom of the trapezium-shaped block face to bind serial-sections into ribbons that were floated onto microscope slides. Ribbons of BMMA serial-sections can be cut using an ultra-microtome and a DiAtome Jumbo-histo diamond knife (DiAtome, Switzerland) with an 8 mm wide blade, which is the limit of the sectioning width. Chloroform vapor was used to remove compression of sections and folds caused by cutting that effect staining and imaging results for 3-D reconstruction. It was possible to cut up to 3 ribbons, with a maximum of 25 serial-sections per ribbon, to 3-D reconstruct 150 µm of tissue per slide.

Polylysine or gelatin-coated slides are required for plastic sections to adhere to the slide and not be removed through heat-mediated antigen retrieval and multiple staining and washing steps. After sectioning, slides are dried at 55 °C on a slide warmer for at least 1 hour. Slides can then be stored indefinitely at room temperature before immuno-labeling and fluorescence imaging.

### Sequential Immuno-staining and 2-D image acquisition

Before immuno-staining, BMMA serial-sections were de-plasticized using acetone and then re-hydrated by immersion in 95%, then 75%, and finally 50% ethanol for 10 minutes each. After re-hydration, serial-sections were washed in 1X PBS (Phosphate buffered-saline) for 5 minutes before heat-mediated antigen retrieval and immuno-labeling.

Heat-mediated antigen retrieval was performed using a pressure cooker in a microwave. Sodium Citrate buffer pH 6 is used and is heated in the pressure cooker to allow it to reach full pressure. Once the pressure cooker is at full pressure, the slides with serial-sections are heated in the pressure cooker for 7 minutes at full pressure. Slides are then left to cool in room temperature 1X PBS before the serial-sections are encircled with a hydrophobic pen, to keep the antibodies within an area around the sections.

Tissue sections were blocked with 5% goat serum for 1 hour before primary antibody staining overnight at 4 °C. Primary antibodies were used at a 1/1000 concentration and included mouse anti-αSMA (Sigma Aldrich – a2547), monoclonal rabbit anti- keratin 5 (Abcam ab52635), keratin 6 (Abcam ab93279) and keratin 8 (Abcam ab53280), polyclonal rabbit anti- keratin 1 (Abcam ab185628), and Ki67 (Abcam ab15580). The rabbit monoclonal antibody against Pparγ was sourced from cell signaling (Cell Signaling - #2435). Slides were washed with 1X PBS three times for 10 minutes to remove unbound primary antibodies, and then secondary antibody staining was performed at 37 °C on a slide warmer for 1 hour away from light. Secondary antibodies used were Anti-rabbit AlexaFluor 568 (Abcam ab175471) and Anti-mouse AlexaFluor 488 (Abcam ab150113), at a concentration of 1/1500. Slides were washed once more in 1X PBS for three times and then mounted in 1 in 5000 DAPI diluted in 1:1 glycerol:1X PBS. Glycerol:PBS mounting agent can be easily removed with the coverslip to perform sequential immuno-staining. Because serial-section ribbons occupy most of the slide, 24 × 50 mm or 24 × 60 mm coverslips are recommended.

Fluorescence imaging was performed using a 20X/0.75NA objective on a Leica DMI6000B, and to capture mosaic images, each image tile was autofocused using the LAX software (Leica, Germany). Pixel size was 0.44 µm (X) x 0.44 µm (Y) with the axial resolution (Z) determined by the section thickness at 2 µm. Mosaic images were stitched at a 10% overlap. To increase image speed, a 160 × 110 mm slide insert with space for 4 slides was used (ASI Imaging, Eugene, OR). Second Harmonic Generation (SHG) imaging was performed using a Zeiss LSM710 equipped with a femtosecond laser tuned to 800 nm. SHG is a non-linear optical effect that enables the imaging of non-centrosymmetric structures, such as fibrillar collagen. Transmission electron microscopy was performed using a JOEL 1010 electron microscope after staining of serial-sections mounted onto grids with 1% uranyl acetate and 0.5% phospho-tungstic acid to improve image contrast. The pixel sizes for SHG and fluorescence images using 20X objective lens attached to a Leica DMI6000B fluorescence microscope were 0.44 μm x 0.44 μm (X-Y). Pixel size for the electron microscope was 0.33 nm x 0.33 nm at X20,000 magnification.

After fluorescence imaging of antibody labeling, serial-sections were eluted of antibodies for sequential immuno-staining by pH 2.0 glycine hydrochloride. Glycine hydrochloride is heated to 55 °C and tissue sections are washed for 15 minutes three times to remove antibodies. Increasing the temperature of the glycine hydrochloride solution can permanently remove DAPI staining.

### 3-D reconstruction, segmentation and quantification of sub-populations

The sequence of high-resolution 2-D images, acquired from immuno-stained BMMA serial-sections, were collated into unaligned image stacks using ImageJ. Unaligned image sections were automatically aligned using Amira software and the ‘Align Slices’ module, so that each DAPI-stained cell nuclei can be aligned with respect to each serial-section in sequence. This generated a 3-D volume of aligned image stacks determined by the number of serial-sections imaged. Therefore, the 3-D voxel size was determined by the objective lens and lateral resolution of the 2-D image (X and Y = 0.44 μm when using the 20X objective on the Leica DMI6000B), and the axial resolution was determined by the physical thickness of each serial-section (Z = 2 μm). The serial-sections were cut at 2 μm as this is the thickness were every cell nucleus lies within the focal plane of the microscope to produce an in-focus image of the entire section, while it reduces the number of sections required for a large volume reconstruction.

Quantification of cell sub-populations based on antibody-tagged proteins or those expressing endogenous fluorescent proteins, such as H2B-GFP fusion protein, was possible by segmenting the cells with positive fluorescent signals in Amira. Segmentation is the assigning of different labels to image voxels that are selected to separate out objects from the 3-D rendered volume. Segmentation can be performed by manual tools to follow the contours of structures or by automated thresholding based on pixel intensity, which was used here to determine the fluorescence intensity of GFP^+^ labelled cells. Using this approach, we qualitatively assessed the fluorescence intensity of each LRC by thresholding the GFP channel between 0–255 and assigned 0–100 as low label-retention, 100–200 as mid label-retention, and 200+ as high GFP label-retention. Importing the segmented mask of each LRC into ImageJ enables 3-D quantification of positively stained cells by using 3-D objects counter, taking the average of three signal thresholds to provide a more accurate mean of cell quantification (high, medium, low).

## Supplementary information

Supplementary Information

Forelimb 3D Movie

Cornea 3D Movie
