# Supplementary material for: Immunofluorescence Tomography: High-resolution 3-D reconstruction by serial-sectioning of methacrylate embedded tissues and alignment of 2-D immunofluorescence images
Source: Sci Rep. 2019 Feb 13;9:1992. doi: 10.1038/s41598-018-38232-9 (PMC6374383; doi:10.1038/s41598-018-38232-9)
Supplement: Supplementary file 1 — Supplementary Information [file 41598_2018_38232_MOESM1_ESM.docx]

**Supplementary information**

**Immunofluorescence Tomography: High-resolution 3-D reconstruction by serial-sectioning of methacrylate embedded tissues and alignment of 2-D immunofluorescence images.**

Geraint J. Parfitt^1,2^

^1^European Cancer Stem Cell Research Institute

^2^School of Optometry & Vision Sciences


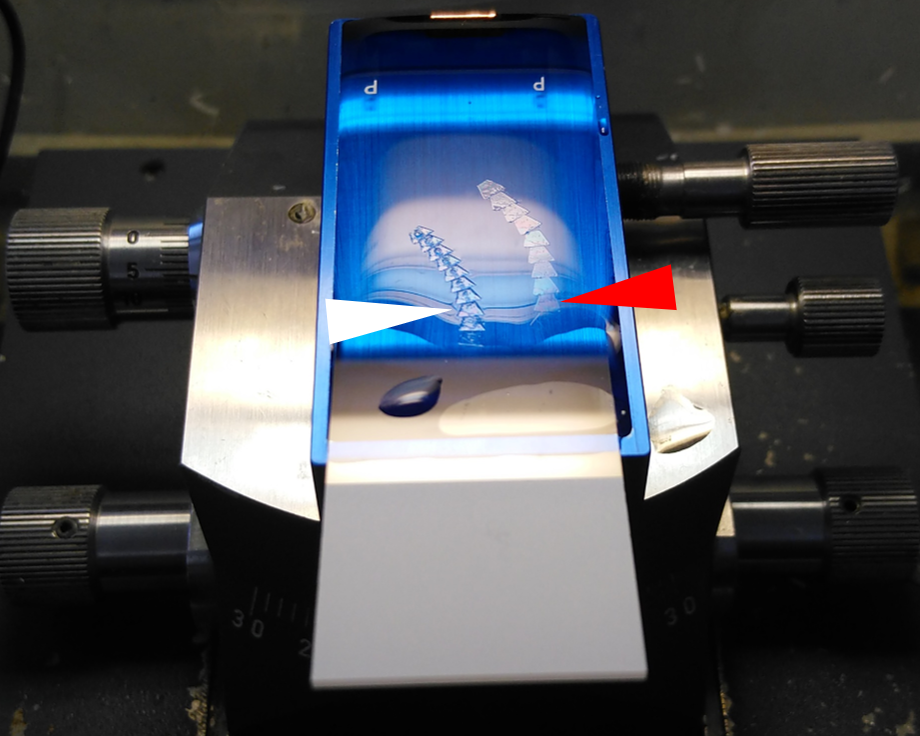


**Supplementary Figure 1: Serial-section ribbons can be removed of compression artifacts by exposure to chloroform vapour. (Red arrow) 2µm Sections exposed to chloroform do not contain folds, unlike (White arrow) serial-sections that are freshly cut.**

**Supplementary Video 1: 3-D reconstruction of keratin 5 expression in the wild-type mouse developing forelimb at E15.5. DAPI staining (green) shows all cell nuclei and keratin 5 marks the superficial epidermis (orange). (1066µm Z)**

**Supplementary Video 2: Immunofluorescence tomography 3-D reconstruction of the mouse cornea. Cell nuclei are labelled with DAPI and epithelial cells are blue, keratocytes and endothelial cells are red. (194µm Z)**
